# Cardiotoxic Effects of Short-Term Doxorubicin Administration: Involvement of Connexin 43 in Calcium Impairment

**DOI:** 10.3390/ijms18102121

**Published:** 2017-10-11

**Authors:** Michela Pecoraro, Antonio Rodríguez-Sinovas, Stefania Marzocco, Michele Ciccarelli, Guido Iaccarino, Aldo Pinto, Ada Popolo

**Affiliations:** 1 Department of Pharmacy, University of Salerno, Fisciano (SA) 84084, Italy; mipecoraro@unisa.it (M.P.); smarzocco@unisa.it (S.M.); pintoal@unisa.it (A.P.); 2 Cardiovascular Diseases Research Group, Department of Cardiology, Vall d’Hebron University Hospital and Research Institute, Universitat Autònoma de Barcelona (UAB), Barcelona 08035, Spain; antonio.rodriguez.sinovas@vhir.org; 3 Department of Medicine and Surgery, University of Salerno, Baronissi (SA) 84084, Italy; mciccarelli@unisa.it (M.C.); giaccarino@unisa.it (G.I.)

**Keywords:** connexin 43, doxorubicin, cardiotoxicity, calcium homeostasis, mitochondria

## Abstract

The use of Doxorubicin (DOXO), a potent antineoplastic agent, is limited by the development of cardiotoxicity. DOXO-induced cardiotoxicity is multifactorial, although alterations in calcium homeostasis, seem to be involved. Since even the Connexin43 (Cx43) plays a pivotal role in these two phenomena, in this study we have analyzed the effects of DOXO on Cx43 expression and localization. Damage caused by anthracyclines on cardiomyocytes is immediate after each injection, in the present study we used a short-term model of DOXO-induced cardiomyopathy. C57BL/6j female mice were randomly divided in groups and injected with DOXO (2 or 10 mg/kg i.p.) for 1–3 or 7 days once every other day. Cardiac function was assessed by Echocardiography. Sarco/endoplasmic reticulum Ca^2+^-ATPase (SERCAII) and phospholamban (PLB) expression were assessed by Western blot analysis, intracellular [Ca^2+^] were detected spectrofluorometrically by means of Fura-2 pentakis (acetoxymethyl) ester (FURA-2AM), and Cx43 and pCx43 expression and localization was analyzed by Western blot and confirmed by immunofluorescence analysis. DOXO induces impairment in Ca^2+^ homeostasis, already evident after a single administration, and affects Cx43 expression and localization. Our data suggest that DOXO-induced alterations in Ca^2+^ homeostasis causes in the cells the induction of compensatory mechanisms until a certain threshold, above which cardiac injury is triggered.

## 1. Introduction

Doxorubicin (DOXO) is a potent broad-spectrum antineoplastic drug, but the development of acute and chronic cardiotoxicity limits its clinical utility [1]. DOXO-induced cardiotoxicity comprise disturbances in cardiac rhythm [2], changes in blood pressure [3], reduction of ejection fraction and contractile function [4], cardiac dilation [5], and cardiomyopathy [6], are often not clinically evident until the late stages. DOXO-induced cardiotoxicity is mainly related to accumulation of the repetitive doses required by the treatments; total DOXO cannot exceed 500 mg/m^2^, but recent evidence indicates that the damage caused by anthracyclines on cardiomyocytes is an early event, already evident after a single administration [7,8].

The exact pathogenesis of DOXO-induced cardiotoxicity is not fully understood even if it is assumed that it is multifactorial [9,10,11,12]. Several lines of evidence indicate that DOXO-induced cardiomyopathy is characterized by abnormal calcium homeostasis, but most of the studies published report only the effects of long-term DOXO-administration [13,14,15,16]. Recently, we have demonstrated that DOXO administration is able to induce calcium dysregulation and Connexin43 (Cx43) re-arrangement in a rat cardiomyoblast cell line already evident after a short-term administration [17]. Cx43 is the most abundant gap junction protein in the heart [18], where it is involved in transcellular electrical conduction and cell survival. In the heart, gap junctions are responsible for the cell-to-cell connection and the propagation of the electrical potential between cardiomyocytes and synchronous contraction [19]. Alterations in Cx43 expression and/or distribution are common features in cardiovascular diseases, i.e., in hypertrophic cardiomyopathy, heart failure and ischemia [20,21,22]. Cx43 is a phosphoprotein and phosphorylation on each of its phosphorylation sites affects membrane insertion and degradation [23,24], and shapes the open probability of the pore [25]. The phosphoproteins arranged in a slightly twisted conformation, highly susceptible to intracellular Ca^2+^, maintain pore patency; increase in intracellular Ca^2+^ results in closure of the pores [26]. Besides its role in plasma membrane, mitochondrial Cx43 (mCx43) has demonstrated a central role in cardioprotection [27]. Cx43 is functionally associated with calcium [28]. Furthermore, it has been demonstrated that Cx43 remodelling might be responsible for intracellular calcium overloading [29], thus resulting in ischemic arrhythmia [30]. As cardiotoxic effects of DOXO imply dysfunction in calcium homeostasis, we hypothesized that DOXO-induced toxicity could also involve Cx43. The aim of this study was to investigate the effects of DOXO administration on Cx43 expression and localization and on calcium homeostasis in a short-term model of DOXO-induced cardiotoxicity in mice.

## 2. Results

### 2.1. Cardiac Functions

Mice were subjected to echocardiography at baseline and before the sacrifice to analyze the main parameters of cardiac function. As reported in Table 1, DOXO administration affects EF, FS, LVEDD, LVESD, and IVSs in mice after a single injection of DOXO. Indeed, compared with control mice, DOXO-treated mice exhibited decreased cardiac systolic function as measured by EF, FS, and IVSD, consequently increased LVEDP and LVESD. 

### 2.2. Doxorubicin Administration Alters Calcium Homeostasis

**Figure 1:**
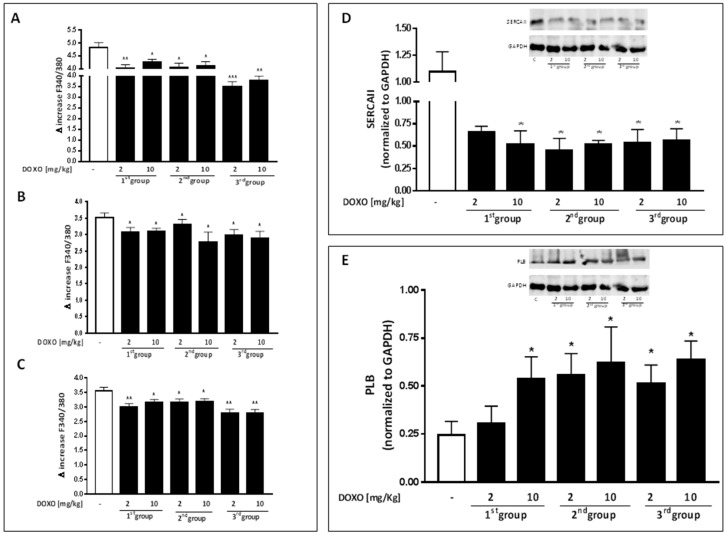
Effect of DOXO on calcium homeostasis. Mice received a single administration (1th group), two administrations (2nd group) or three administrations (3rd group) of DOXO (2 or 10 mg/kg; i.p.) and primary cardiomyocytes were isolated by enzymatic digestion. Intracellular calcium content in cells suspension was evaluated by using ionomycin (1 μM) (**A**); reticulum calcium content was evaluated by means of thapsygargin (100 nM) (**B**) and mitochondrial calcium content was evaluated by using FCCP (50 nM) (**C**). Results were expressed as mean ± S.E.M. of delta (δ) increase of FURA-2 AM ratio fluorescence (340/380 nm) from at least three independent experiments each performed in duplicate. Data were analyzed by Student’s *t*-test. * *p* < 0.05, ** *p* < 0.005 and *** *p* < 0.001 vs. control. Effect of DOXO on SERCA II (**D**) and PLB (**E**) expression. Mice received a single administration (first group), two administrations (second group), or three administrations (third group) of DOXO (2 mg/kg or 10 mg/kg; i.p.) and SERCA II and PLB expressions were detected by Western blot analysis into tissue homogenates from mice; GAPDH protein expression was used as loading control. Values were expressed as mean ± S.E.M. from at least three independent experiments each performed in duplicate. Data were analyzed by Student’s *t*-test. *p < 0.05 vs. control.

It is well known that impairment in [Ca^2+^]_i_ plays a pivotal role both in cardiotoxic activity of DOXO [1] and in Cx43 activity [29]. In order to evaluate the effects of DOXO treatment on intracellular Ca^2+^ levels in our experimental model, primary cardiomyocytes from hearts of DOXO-treated mice and from control mice were isolated and loaded with the fluorescent dye FURA2-AM in Ca^2+^-free incubation medium (containing 0.5 mM EGTA). Our data indicate that DOXO treatment increased basal level of [Ca^2+^]_i_. Indeed the delta increase in [Ca^2+^]_i_ induced by ionomycin, a Ca^2+^ ionophore, in cardiomyocytes from DOXO-treated mice was significantly (*p* < 0.05) lower than that found in cells from control mice at all experimental time points (Figure 1A). An increase in basal Ca^2+^ levels may indicate that the cells are not capable of storing Ca^2+^ in intracellular stores. Accordingly (Figure 1B), delta increase in [Ca^2+^]_i_ induced by thapsigargin, an endoplasmatic reticulum Ca^2+^ stores depletor, in cardiomyocytes from DOXO-treated mice was significantly lower (*p* < 0.001) than that registered in cardiomyocytes from control mice, even in presence of a single DOXO administration, which may indicate a reduced Ca^2+^ storage in the endoplasmic reticulum. In the same way, the delta increase in [Ca^2+^]_i_ induced by FCCP, a mitochondrial Ca^2+^ depletory, in cardiomyocytes from DOXO-treated mice was significantly (*p* < 0.001) lower than that found in cells from control mice in all experimental groups (Figure 1C), suggestive of less build-up of Ca^2+^ also in the mitochondria.

Among all regulatory mechanisms involved in intracellular Ca^2+^ homeostasis, SERCAII plays a pivotal role. Many studies report how changes in the expression and/or activity of SERCAII, regulated by phospholamban (PLB), are altered in many forms of cardiomyopathy [31]. Therefore, we analyzed the expression levels of SERCAII and PLB and Ca^2+^ levels in the heart of DOXO-treated mice. Data obtained by Western blot analysis showed a significant (*p* < 0.05) reduction of SERCAII expression always in mice that received a single DOXO administration (Figure 1D) and a concomitant increase in PLB expression (Figure 1E).

### 2.3. Doxorubicin Administration Affects Cx43 Expression and Localization

**Figure 2:**
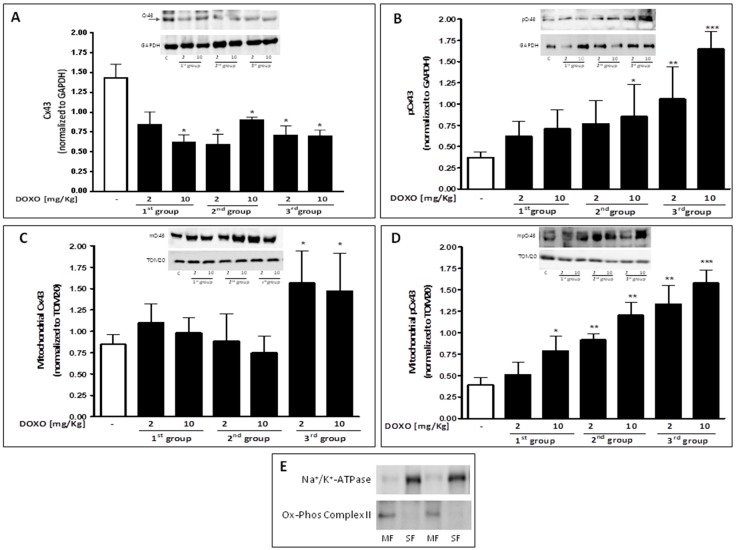
Mice received a single administration (first group), two administrations (second group), or three administrations (third group) of DOXO (2 mg/kg and 10 mg/kg; i.p.) and Cx43 and pCx43 expressions were detected by Western blot analysis into tissue homogenates from mice; GAPDH protein expression was used as loading control (**A**,**B**); Effects of DOXO on mCx43 (**C**) and mpCx43 (**D**) expression were detected by Western blot analysis on mitochondrial protein lysate from mice; TOM20 protein expression was used as loading control. Values were expressed as mean ± S.E.M. from at least three independent experiments each performed in duplicate. Values are expressed as mean ± S.E.M. from at least three independent experiments each performed in duplicate. Data were analyzed by Student’s *t*-test. * *p* < 0.05, ** *p* < 0.005 and *** *p* < 0.001 versus control. Representative Western blots of Na^+^/K^+^ ATPase and Ox-Phos Complex II were used as markers of sarcolemma (SF) and mitochondria (MF), respectively, to demonstrate the purity of the mitochondrial extracts (**E**).

Besides alterations in Ca^2+^ homeostasis, several forms of cardiomyopathy, such as hypertrophy, and dilated and ischemic cardiomyopathy are also characterized by abnormal Cx43 expression and distribution in the heart [32]. As depicted in Figure 2A, DOXO-treatment induces a decrease of total Cx43 also evident in mice that received a single dose of DOXO.

Western blot analysis performed on heart lysates of treated-mice confirmed that, compared to control mice, DOXO-treated mice had a significant (*p* < 0.05) reduction of Cx43 expression, that could be observed in all experimental groups (Figure 2A). Phosphorylation of Cx43 affects its main biological properties [33]. In particular, phosphorylation on Ser368 is linked to metabolic and/or electrical uncoupling of gap junctions [34] and induces channels closure [35]. Accordingly, Western blot analysis showed a significant (*p* < 0.05) increase in Cx43 phosphorylated on Ser368 in DOXO-treated mice (Figure 2B).

**Figure 3:**
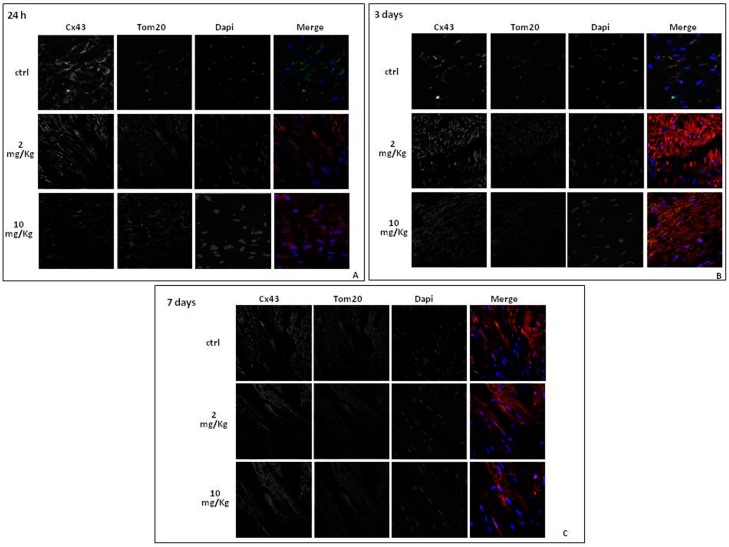
Effect of DOXO (2 and 10 mg/kg; i.p.) on Cx43 localization in heart of C57BL/6j mice which received a single administration (first group), two administrations (second group), or three administrations (third group). Frozen myocardial tissue sections were stained with Anti-Cx43 (green), TOM20 (red) and nucleus with DAPI (blue) and were determined by Immunofluorescence assay at confocal microscopy for mitochondrial Cx43 localization. Scale bar 10 µm.

In addition to the function performed at gap junction levels, it has recently been shown that Cx43 is also expressed at mitochondrial level, where it is involved in cardioprotection [36,37]. Mitochondrial Cx43 levels increase in stress conditions such as ischemia-reperfusion [27] or DOXO-induced cardiotoxicity [17]. Western blot analysis on mitochondrial lysates from hearts of DOXO-treated mice confirmed that Cx43 expression on mitochondria significantly (*p* < 0.05) increases with the treatment duration in DOXO-treated mice (Figure 2C). Mitochondrial expression of Cx43 phosphorylated on Ser368 significantly (*p* < 0.005) increased in a time dependent-manner in the heart of DOXO-treated mice (Figure 2D). Immunofluorescence analysis performed on heart sections double-stained for Cx43 and TOM20, as a marker of mitochondria, confirmed an increased mitochondrial localization of Cx43 in the heart of DOXO-treated mice (Figure 3). 

## 3. Discussion

The anthracycline anticancer drug DOXO is a cornerstone in many malignancies. However, its clinical use is hindered by high risk of cardiotoxicity [38]. Much is known about the long-term toxicity of DOXO, but just as important appear to be its short-term effects [8,39]. DOXO-induced acute cardiotoxicity occurs in about 11% of patients in which the onset of paroxysmal non sustained supraventricular tachycardia and premature atrial and ventricular beats are observed [4]. The mechanisms underlying DOXO cardiotoxicity are not fully understood, but a large body of evidence indicates that anthracyclines interfere with calcium homeostasis, that is largely involved both in cardiac dysfunction and in apoptosis, the two major signs of DOXO-induced cardiotoxicity. In view of the pivotal role played by Cx43 in cardiac function and in cell death signal propagation, this study aimed to analyze the effect of DOXO administration on Cx43 expression and localization in a short-term model of DOXO-induced cardiotoxicity in mice developed by our group and previously published [8]. Doses of Doxo administered are 2 and 10 mg/kg and are the lowest doses reported in the literature [40,41].

It is worth noting that the used doses, although lower than those used in humans or in other animal studies, are able to induce clear signs of cardiac dysfunction, such as decreased EF and FS and increased LVESD, and are even evident in mice that received a single administration of DOXO. It is well known that systolic and diastolic function of the heart are regulated by calcium handling [42]. In our experimental model, we observed a significant dysfunction in Ca^2+^ homeostasis in DOXO-treated mice. Indeed, as confirmed by means of Fura-2 pentakis(acetoxymethyl) ester (FURA-2AM), endoplasmic reticulum Ca^2+^ content from cardiomyocytes isolated from DOXO-treated mice was significantly lower than that of control mice at all experimental times. In line with earlier reports [31], Western blot analysis showed a significant decrease of SERCAII expression in the heart of DOXO-treated mice, confirming the central role of SERCAII in maintaining intracellular calcium homeostasis in the heart [42]. Moreover, our results showed a significant increased expression of PLB, a protein expressed in the sarcoplasmic reticulum membrane with inhibitory effects on SERCAII, in DOXO-treated mice, with a further alteration of cytosolic Ca^2+^ content. Previous studies suggest a functional role of the gap junction/intercellular communication in the regulation of Ca^2+^ signalling in diseased heart [43,44], which may explain, at least in part, our findings on intracellular Ca^2+^ regulation. Abnormal expression of Cx43, the main gap junction’s protein in the heart, has been reported in several forms of cardiomyopathies: i.e., hypertrophic, dilated, and ischemic cardiomyopathy. Furthermore, recently alterations in Cx43 expression and localization have been proved in DOXO-treated cardiomyoblast cell line [17]. Our data demonstrate that, as in other forms of cardiomyopathies, in our experimental model of DOXO-induced acute cardiomyopathy, Cx43 expression was significantly reduced.

Downregulation and heterogeneous redistribution of Cx43 in diseased heart has been reported [32]. In this study we demonstrated that in addition to reducing expression of Cx43, DOXO significantly increases the expression of Cx43 phosporylated on Ser368. PKC-mediate phosphorylation on Ser affects the metabolic and electrical conduction through the gap junction. Specifically, phosphorylation on Ser368 creates a ‘closed’, conformational state, with a consequent electrical and chemical uncoupling. Furthermore, phosphorylation on Ser368 blocks the access of other kinases and phosphatases to the C-terminal residue [45]. It has been reported that interruption of cell-to-cell communication through gap junctions is a mechanism of defense implemented by cells to block the propagation of harmful stimuli. This event takes the name of “good Samaritan” effect [46] even if this involves alterations in the propagation of electrical impulses. So we can hypothesize that the reduced expression of Cx43 and the concomitant increase of Cx43 phosphorylated on Ser368 in the heart of DOXO-treated mice is a mechanism put in place to try to defend the cardiomyocytes from the propagation of harmful stimuli, such as calcium overload, although this involves alterations in cardiac functions such as those observed with echocardiography in our experimental model. 

Intracellular Ca^2+^ homeostasis is fine-tuned by the cyclical uptake and release by the different cellular compartments [47]. Recent evidence points to a role of Cx43 in mitochondrial homeostasis of Ca^2+^ [48,49]. 

The increase of mCx43 in cardiomyocytes can be induced by various stimuli, such as cellular stress and ischemic preconditioning, but its functional relevance is still unclear. It has been postulated that mCx43 is part of multiprotein complex that somehow controls mitochondrial homeostasis and that it forms hemichannels that serve as a conduit for ion flux [50], like Ca^2+^. Interaction between proteins involved in this multiprotein complex is reinforced by PKC that mediates phosphorylation of the Cx43 at Ser368 residue [51]. In our experimental model, we found a significant increase of mCx43 and of mCx43 phosporylated on Ser368 expression. mCx43 protects cardiomyocytes by mitigating Ca^2+^ overload, mitochondrial permeability transition, and cell death [47]. In agreement with this hypothesis, our data show that increased mCx43 and mCx43 phosphorylated on Ser368 expression is associated with a reduced accumulation of Ca^2+^ in the mitochondria. 

## 4. Materials and Methods 

### 4.1. Materials

DOXO was purchased from Baxter manufacturing S.p.a. (Officina di Sesto Fiorentino, Florence, Italy). Where not indicated otherwise, antibodies used were purchased from Santa Cruz Biotechnology (DBA, Milan, Italy), and all other products were purchased from Sigma (Milan, Italy). 

### 4.2. Animals

Six week old female C57BL/6j (weighting 20–22 g) were purchased from Charles River (Lecco, Italy). All experimental procedures that involve animals have been conducted in agreement with the Italian and European Community Council for Animal Care (DL. no. 26/2014 protocol number of Ministerial approvation DGSAF 13788-A 02/07/2015) and in accordance with the guidelines of the Guide for the Care and Use of Laboratory Animals of the National Institutes of Health. 

### 4.3. Experimental Protocols

This study was performed using a previously well-established animal model of short-term DOXO induced cardiotoxicity [8]. Briefly, C57BL/6j mice were randomly divided in three groups (*n* = 6 for each experimental group). The doses used were 2 and 10 mg/kg [40,41] and they corresponded to 6.142 and 30.8 mg/m^2^ respectively [52].

First Group: received one DOXO administration (2 or 10 mg/kg i.p.) and were sacrificed 24 h after the treatment;

Second Group: received two DOXO administrations (2 or 10 mg/kg i.p.) once a day for two days and were sacrificed three days after the first administration;

Third Group: received three DOXO administrations (2 or 10 mg/kg i.p.) once a day for three days and were sacrificed seven days after the first administration.

Mice that received saline were used as control group. Cardiac function was monitored echocardiographically at baseline and before sacrifice. At the end of each experimental time, heart samples were collected and prepared for molecular biological analyses.

### 4.4. Echocardiogram

Mice were lightly anesthetized with 1–1.5% of isoflurane in oxygen until the heart rate stabilized to 400–500 beats per minute. Echocardiography was performed using VEVO (VisualSonic, Toronto, ON, Canada) instrument. Ejection fraction (EF), Fractional shortening (FS), Left Ventricular End-Diastolic-Diameter (LVEDD), and Left Ventricular End-Systolic Diameter (LVESD) were calculated using the VEVO analysis software.

### 4.5. Protein Extraction and Western Blot Analysis 

Total proteins were extracted by homogenization of hearts with a dounce potter in lysis buffer (TRIS-HCl 50 mM, NaCl 500 mM, protease inhibitors, PMSF 0.25 μM, NaF 50 mM, Na_3_VO_4_ 0.2 mM). Protein concentrations were determined with the Bio-Rad protein assay (BIO-RAD, Milan, Italy). Equal amounts of protein (50 µg) were loaded into an acrylamide gel and separated by SDS-PAGE under denaturating conditions. Blots were incubated with primary antibody anti-Cx43 (Sigma C6219), anti-pCx43 phosphorylate on Ser368 (pCx43; Santa Cruz, Dallas, TX, USA, SC-17219-R), anti-sarco/endoplasmic reticulum Ca^2+^-ATPase (SERCAII; Santa Cruz: SC-376235), anti-phospholamban (PLB; Santa Cruz: SC-21923), or anti-glyceraldehyde 3-phosphate dehydrogenase (GAPDH; Santa Cruz: 32233) (used as loading control) overnight at 4 °C. After incubation period with the primary antibodies, blots were washed in PBS 0.1% Tween. The appropriate secondary antibody—anti-rabbit, anti-mouse, or anti-goat (each diluted 1:4000)—was added and allowed in incubation for 1 h at room temperature. Immunoreactive protein bands were detected by chemiluminescence using enhanced chemiluminescence reagents (ECL) in LAS 4000 (GE Healthcare, (Björkgatan, Uppsala, Sweden).

### 4.6. Mitochondrial Protein Extraction and Western Blot Analysis for Mitochondrial Cx43 and pCx43 

Mitochondrial proteins were extracted from homogenized hearts with a dounce potter in lysis buffer (sucrose 250 mM, K^+^ Hepes pH 7.5 20 mM, KCl 10 mM, MgCl_2_ 1.5 mM, EDTA 0.1 mM, EGTA 1 mM, protease inhibitors, NaF 50 mM, Na_3_VO_4_ 0.2 mM, PMSF 100 μM, DTT 1 mM, digitonin 0.025%) as previously reported [17,53,54]. The protein yield was quantified with the Bio-Rad protein assay (BIO-RAD, Milan, Italy). Western blot analysis for Cx43 or pCx43 was performed as described above. Primary antibody anti-TOM20 (Santa Cruz: SC-11415) was used as loading control. In order to verify the purity of mitochondrial protein extraction, a Western blot analysis was performed to evaluate the presence of proteins expressed only in the mitochondria (ox-Phos Complex II, Abcam, Cambridge, UK: ab14715) and the absence of proteins expressed in other cellular compartments (Na^+^/K^+^ ATPase, Abcam: ab7671) [55].

### 4.7. Primary Cardiomyocytes Isolation and Measurement of Intracellular Ca^2+^ Signaling

Intracellular Ca^2+^ concentrations were measured in primary cardiomyocytes, isolated from hearts of DOXO-treated mice and control mice. Hearts were washed with HBSS 0.1 mM Ca^2+^ (140 mM NaCl, 5.4 mM KCl, 0.44 mM KH_2_PO_4_, 0.42 mM Na_2_H PO_4_, 4.17 mM NaHCO_3_, 26 mM CaNa-EDTA, 0.10 mM CaCl_2_·H_2_O, 5.0 mM HEPES and 5.5 mM dextrose). Next, hearts were cut in 1–2 mm sections and incubated at 37 °C in HBSS 0.1 mM Ca^2+^ containing albumin 10 mg/mL, tryspin inhibitor 1 mg/mL, taurine 5 mM, dithiothreitol 0.4 mg/mL, collagenase II 0.6 mg/mL, and papain 0.6 mg/mL for 75 min. After the incubation period, tissue fragments were removed by filtering the suspension with a 0.70 µ filter. Filtrate was centrifuged to collect the cardiomyocytes. Primary cardiomyocytes were loaded with the ratiometric fluorescent indicator dye FURA2-AM (5 µM, 45 min, 37 °C) at a cell density of 3 × 10^4^ cells/mL in HBSS 0.1 mM Ca^2+^. FURA2-AM excess was removed by washing the cells with HBSS and cardiomyocytes were then transferred to a spectrofluorimeter (Perkin-Elmer LS-50, Waltham, MA, USA). As reported for FURA2-AM, the excitation wavelength was alternated between 340 and 380 nm, and the emission wavelength was 515 nm. The basal F340/F380 ratio was recorded and then treatment with ionomycin (1 µM), a calcium ionophore; thapsigargin (1 µM), an inhibitor of sarco (endo) plasmic reticulum; or carbonyl cyanide p-trifluoromethoxypyhenylhydrazone (FCCP; 50 nM), a mitochondrial calcium depletory, was added into the cuvette in Ca^2+^-free HBSS and F340/F380 ratio was recorded 5 min after each stimulus induced. The ratio of fluorescence intensity of 340/380 nm (F340/F380) is strictly related to intracellular free [Ca^2+^] [56]. Results are expressed as delta increase of F340/F380, calculated as F340/F380 stimulus—F340/F380 basal.

### 4.8. Immunohistochemical Analysis 

For immunohistochemical analyses, frozen cardiac tissues were embedded in OCT compound (Bio-Optica, Milan, Italy). Sections (7 µm) were incubated with mouse anti-Cx43 and rabbit anti-TOM20 for 2 h at room temperature. Then the slides were washed three times with PBS and incubated with secondary antibodies (FITC-conjugated anti mouse IgG and Texas red-conjugated anti rabbit IgG) for 1 h. DAPI was used to mark the nuclei. After mounting, coverslips were examined by using a Laser Confocal Microscope (Leica TCS SP5, Wetzlar, Germany). 

### 4.9. Statistical Analysis

Data are reported as the mean ± standard error mean (S.E.M.) of at least three independent experiments. Statistical differences were assessed with Student’s *t*-test. *p*-values of less than 0.05 was considered significant.

## 5. Conclusions

In this work, we have shown that even a short-term administration of DOXO is able to induce significant changes in calcium homeostasis and alterations in Cx43 expression and localization. These effects are evident even in hearts of mice that received a single DOXO-administration, but such a rapid response is not surprising. Indeed, each DOXO administration can induce structural changes in cardiomyocytes that ultimately lead to death of cardiomyocytes themselves. These alterations may be somewhat balanced by the establishment of compensatory mechanisms until a certain threshold, above which cardiac injury is triggered [57].

## Figures and Tables

**Table 1:** Effect of Doxorubicin DOXO (2 or 10 mg/kg; i.p.) on left ventricular end diastolic diameter (LVEDD), left ventricular end systolic diameter (LVESD), ejection fraction (% EF), and fraction shortening (% FS) after a single administration (1st group), two administrations (2nd group), or three administrations (3rd group). Results were expressed as mean ± S.E.M. from 6 mice/group.

Groups	Parameters	Control	2 mg/kg	10 mg/kg
1st group	LVEDD	3.97 ± 0.11	3.92 ± 0.11	4.09 ± 0.10
	LVESD	2.62 ± 0.17	2.77 ± 0.09	3.00 ± 0.06 *
	% EF	62.17 ± 4.1	58.39 ± 1.12 *	52.7 ± 1.38 **
	% FS	30.41 ± 0.72	33.26 ± 2.93	26.76 ± 0.92 *
2nd group	LVEDD	3.94 ± 0.05	3.87 ± 0.05	3.99 ± 0.06
	LVESD	2.78 ± 0.054	2.78 ± 0.05	2.90 ± 0.06*
	% EF	57.2 ± 1.25	54063 ± 1.6 *	53.73 ± 1.61 *
	% FS	30.41 ± 0.85	27.95 ± 1.06 *	27.43 ± 1.02 *
3rd group	LVEDD	3.86 ± 0.04	3.94 ± 0.07	3.96 ± 0.06 *
	LVESD	2.73 ± 0.15	2.85 ± 0.06	2.9 ± 0.14 *
	% EF	59.00 ± 4.17	54.4 ± 2.6 *	50.49 ± 4.79 **
	% FS	31.76 ± 1.68	30.97 ± 2.91	25.37 ± 3.02 *
